# Effects of Defatted Black Soldier Fly (*Hermetia illucens*) Meal on the Performance, Digestibility and Blood Parameters of Weaned Piglets

**DOI:** 10.3390/ani16111571

**Published:** 2026-05-22

**Authors:** Mara Parreiras, Victor Pinheiro, Olga Moreira, Maria Soares, Daniel Murta, Ana Novo Barros, Divanildo Outor-Monteiro

**Affiliations:** 1 Department of Animal Sciences, University of Trás-os-Montes and Alto Douro (UTAD), 5000-081 Vila Real, Portugal; vpinheir@utad.pt (V.P.); divanildo@utad.pt (D.O.-M.); 2 Center for Animal and Veterinary Science (CECAV), University of Trás-os-Montes and Alto Douro (UTAD), 5000-081 Vila Real, Portugal; 3 National Institute for Agricultural and Veterinary Research, Fonte-Boa, 2005-048 Santarém, Portugal; olga.moreira@iniav.pt; 4 Associated Laboratory for Animal and Veterinary Science (Al4Animals), Faculdade de Medicina Veterinária da Universidade de Lisboa, 1300-477 Lisbon, Portugal; msoares@egasmoniz.edu.pt; 5 Moniz Center for Interdisciplinary Research (CiiEM), Egas Moniz School of Health & Science, Campus Universitário, Quinta da Granja, Caparica, 2829-511 Almada, Portugal; dmurta@egasmoniz.edu.pt; 6 Department of Chemistry, University of Trás-os-Montes and Alto Douro (UTAD), 5000-081 Vila Real, Portugal; abarros@utad.pt; 7 Center for Research and Agro-Environmental and Biological Technologies (CITAB), University of Trás-os-Montes and Alto Douro (UTAD), 5000-081 Vila Real, Portugal

**Keywords:** defatted BSF larval meal, weaned piglets, growth performance, digestibility, blood profile

## Abstract

This study investigated the effects of different inclusion levels of defatted black soldier fly (*Hermetia illucens*: BSF) larval meal on growth performance, haematological and biochemical blood parameters, and nutrient digestibility in piglets. Forty-eight male piglets weaned at 28 days of age ((Landrace × Large White) × (Piétrain)) were randomly assigned to three experimental treatments. Each treatment included eight pens with two piglets per pen. Diets were formulated to be isoenergetic and isoproteic, containing defatted BSF meal as a partial replacement for fishmeal and soybean meal: Control (0% BSF), BSF3% (3% inclusion), and BSF6% (6% inclusion). Over the whole experimental period, dietary treatment did not significantly affect final body weight, average daily gain, average daily feed intake, or feed conversion ratio, although feed intake differed during the 7–28-day period. Red and white blood cell parameters were not influenced by diet. Apparent total tract digestibility of dry matter and organic matter increased at the 6% inclusion level, while crude protein and crude fat digestibility were not affected. These results indicate that defatted BSF meal can be used as a sustainable alternative protein source in piglet diets, maintaining growth performance and health status.

## Simple Summary

This study assessed the replacement of fishmeal and soybean meal with defatted black soldier fly (*Hermetia illucens*; BSF) larval meal in piglet diets. Forty-eight weaned piglets were allocated to three dietary treatments containing 0%, 3%, or 6% defatted BSF meal. Growth performance, blood parameters and nutrient digestibility were measured. Inclusion of BSF meal up to 6% did not affect average daily gain, feed intake, or feed conversion ratio in the total period of trial. Haematological values remained within normal physiological ranges. Apparent total tract digestibility of dry matter and organic matter increased at the 6% inclusion level, while crude protein and crude fat digestibility were unaffected. These results suggest that defatted black soldier fly larvae meal can be incorporated at up to 6% in weaned piglet diets without adverse effects on performance or health-related blood variables.

## 1. Introduction

Soybean meal is currently the main protein source used in animal feed because of its high protein content and favourable amino acid profile [1,2,3,4,5]. However, the increasing global demand for soybean has contributed to environmental concerns such as deforestation and biodiversity loss, intensifying the search for more sustainable alternative protein sources [6,7,8,9,10]. Fishmeal is also an important protein source due to its high-quality protein and omega-3 fatty acids [11,12]. Nevertheless, overfishing and increasing costs have raised concerns regarding its long-term sustainability [13]. Consequently, there is growing interest in identifying alternative and more sustainable protein sources for livestock production.

Among these alternatives, insects have emerged as a promising option, particularly the black soldier fly (*Hermetia illucens*) [14,15,16]. Black soldier fly (BSF) larvae contain high levels of protein and present an amino acid profile comparable to soybean meal and fishmeal [2,3,17]. In addition, they are rich in lipids, vitamins, minerals, and bioactive compounds such as antimicrobial peptides, chitin, and lauric acid [2,18,19,20,21,22]. These compounds may positively influence animal health. BSF larvae can also efficiently convert organic waste into high-quality biomass [23,24,25,26,27]. This characteristic contributes to waste reduction and supports circular economy strategies [16,28]. Furthermore, BSF production requires less land and water than conventional protein production systems [19,29,30].

The post-weaning period is a critical stage in pig production. During this phase, piglets are particularly vulnerable to nutritional and physiological stress. Previous studies have shown that the inclusion of BSF larvae meal in piglet diets may improve growth performance, gut health, and immune response [1,31,32,33]. Defatted BSF larvae meal has also demonstrated good nutrient digestibility and tolerance in weaned piglets [34,35]. These beneficial effects are mainly attributed to its balanced nutritional composition and the presence of functional bioactive compounds [2,6,36,37].

Despite the growing interest in the use of BSF larvae meal in pig nutrition, information regarding suitable inclusion levels and their effects on piglet health and nutrient digestibility during the post-weaning phase remains limited.

Therefore, the present study aimed to evaluate the effects of including 3% and 6% defatted BSF meal in diets for weaned piglets, partially replacing soybean meal and fishmeal. Growth performance, haematological and blood biochemical parameters, and apparent digestibility were evaluated.

## 2. Materials and Methods

The experimental procedures were approved by the Animal Welfare Body of the University of Trás-os-Montes and Alto Douro, Portugal, under case number 2253-e-DZ-2022. The study was conducted in accordance with Portuguese Decree-Law No. 113/7 August 2013, which implements Directive 2010/63/EU on the protection of animals used for scientific purposes.

### 2.1. Animals and Experimental Set-Up

The experiment was conducted at Experimental Pig Farming (UTAD BioLab Sus) of the University of Trás-os-Montes and Alto Douro in Portugal.

A total of 48 male piglets ((Landrace × Large White) × (Piétrain)) were weaned at 28 days of age, weighed individually (average body weight of 7.62 ± 0.66 kg) and randomly assigned to three experimental treatments. The piglets were housed in pairs in 24 pens (8 pens and 16 animals per treatment).

Each pen had an area of 2 m^2^ and contained a feeder and a drinker, with the water flow adjusted as the piglets grew. The animals had one week to acclimatise to the feed, management and facilities. At the beginning of the trial, each pen was labelled with a number and the type of feed, and the animals were individually identified with a numbered ear tag.

The dietary treatments were Control, with no defatted black soldier fly meal; BSF3, containing 3% defatted black soldier fly meal; and BSF6, containing 6% defatted black soldier fly meal. The inclusion levels of 3% and 6% defatted *Hermetia illucens* meal were based on previously reported safe inclusion ranges in pig nutrition studies. Experimental evidence in piglets indicates that low to moderate dietary inclusion of *Hermetia illucens* meal does not adversely affect growth performance or nutrient utilisation, as reported by [11,33]. In addition, general recommendations on the use of insects as feed ingredients are provided in the FAO report by [6], which supports their application within safe inclusion ranges in animal nutrition. Based on this evidence, the selected inclusion levels were used to establish a graded substitution strategy within nutritionally balanced and experimentally validated limits. The diets were formulated to be isoenergetic and isoproteic. Piglets were adapted for a week to the housing conditions, feeding system, and experimental diets before data collection began. The feeding programme lasted 6 weeks and included a prestarter diet (from day 1 to day 21) followed by a starter diet (from day 22 to day 49 of the experiment).

### 2.2. Production of Black Soldier Fly Larvae

Black soldier fly larvae were produced at the EntoGreen pilot facility in Santarém, Portugal. Larvae were reared on a substrate composed of locally sourced vegetable by-products, mainly derived from olive oil production, including olive pomace. Rearing was conducted in 40 × 20 cm boxes containing approximately 12 kg of substrate for 14 days, under temperatures ranging from 24 to 38 °C. At the end of the rearing period, larvae were separated from the residual substrate by sieving, dried, and mechanically pressed for lipid extraction. The resulting defatted larvae meal was packed and delivered to the feed manufacturer for diet preparation.

### 2.3. Zootechnical Parameters

The individual body weight of the piglets was recorded weekly. These weights were used to calculate the average daily weight gain (ADG). The feed given to the animals and the portions not consumed were also weighed weekly to calculate the average daily feed intake (ADFI). Body weight gain and the amount of feed consumed were used to calculate feed conversion ratio (FCR).

Faecal consistency was assessed daily in each pen using a four-point scoring system: 1 = normal, 2 = pasty, 3 = semi-liquid, and 4 = liquid. The occurrence of diarrhea was then summarised by week and for the overall experimental period.

### 2.4. Digestibility Trial

An indigestible marker (chromium oxide) was homogeneously mixed into all experimental diets (0.2% chromium oxide) for three days before the faeces collection period. Faeces samples were collected over 5 consecutive days, twice daily (09:00 and 15:00). The faeces collected from each park were weighed and dried daily in an oven at 50 °C until a constant weight was reached. They were then ground in a mill with a 1 mm sieve, and a composite sample was prepared. The faeces and diet samples were analysed to determine dry matter (DM), organic matter (OM), ash, crude protein (CP), crude fat (CF) and neutral detergent fibre (NDF) values according to the AOAC methodologies described. The concentration of chromium in the feed and faeces was used to determine the total amount of faeces and estimate digestibility values according to the following method.

Using the analysed values for nutrient concentration, as well as the concentration of chromium oxide in the feed and faeces, nutrient digestibility was calculated as follows:

$$Digestibility\;\left(\%\right)=100-(100\times \frac{Nf\times Td}{Nd\times Tf})$$

where *Nf* and *Nd* are the nutrient concentrations (% DM) in the faeces and diet, respectively, and *Td* and *Tf* are the Cr_2_O_3_ concentrations (% DM) in the diets and faeces, respectively.

### 2.5. Chemical Analyses, Minerals and Amino Acids

All samples (feed and faeces) were dried at 50 °C to a constant weight in a convection oven (Venticell, MMM Group, Munich, Germany). Samples were ground on a 1 mm sieve (Tecator Cyclotec 1093 Sample Mill, Foss SA, Hoganas, Sweden) and prepared for chemical analysis. AOAC methods were used [38] to determine dry matter (No. 934.01), organic matter, crude ash (No. 942.05) as well as ether extract (CF, No. 920.39) and total nitrogen (N) by Kjeldahl method (954.01) was used. The crude protein content was calculated as N × 6.25, according to the Kjeldahl method. The neutral detergent fibre content was determined without sodium sulphite or α-amylase, following the methods proposed Van Soest et al., [39]. All analyses were performed in duplicate. The centesimal composition of the BSF larvae meal and the experimental diets is shown in Table 1.

The mineral composition of food and feed samples was determined using complementary techniques of X-ray energy dispersive spectroscopy coupled with scanning electron microscopy (SEM-EDS) and X-ray fluorescence spectroscopy (XRF), as described in validated laboratory protocols and specialised literature. The samples were previously dried in an oven at 60 °C until constant mass, aiming at removing free water and stabilising the matrix, and subsequently ground and homogenised to ensure representativeness of the aliquots. For SEM-EDS, small portions of the samples were mounted on conductive supports and covered with a thin layer of carbon to minimise charging effects and optimise electrical conductivity. The analyses were performed under vacuum using controlled electron acceleration conditions, allowing qualitative identification and semi-quantitative quantification of elements such as calcium, phosphorus, potassium, sodium, magnesium, iron, copper, manganese, and zinc. At the same time, the pressed pellets of the samples were subjected to XRF analysis, and the emitted fluorescence was converted into absolute concentrations by calibration with certified standards, following recommendations from recognised reference materials.

The amino acid composition in food and feed samples was determined using the AccQ·Tag™ Ultra (Waters Corporation, Westervoortsedijk 60, Arnhem, 6827-AT, The Netherlands) methodology with high-performance liquid chromatography (HPLC) and fluorescence detection. The samples were homogenized, ground, and a 50 mg aliquot was subjected to acid hydrolysis with 6 N HCl at 110 °C for 24 h to release the protein-bound amino acids. After cooling, the solutions were neutralized and filtered. The released amino acids were derivatized with AccQ·Tag™ Ultra reagent in borate buffer and incubated at 55 °C for 10 min, forming fluorescent derivatives. Chromatographic analysis was performed on a C18 AccQ·Tag™ Ultra column, using a gradient of water with 0.1% trifluoroacetic acid and acetonitrile, with fluorescence detection. Quantification was obtained by comparison with standard curves of known amino acids, expressing the results in mg/g or % dry mass. This procedure allows for the simultaneous analysis of essential and non-essential amino acids, following recommendations established by Waters Corporation, and the official (AOAC 994.12) method [9], ensuring reliable and reproducible results for the nutritional evaluation of foods and feeds.

### 2.6. Blood Collection and Analysis

Blood samples were collected at the end of the piglets’ growth phase (77 days of age) from 48 animals. Two blood samples (5 mL each) were taken from the jugular vein of each piglet into two tubes. One sample was treated with ethylenediaminetetraacetic acid anticoagulant (K2EDTA) for blood cell counts, and the other with a coagulation activator for serum biochemistry.

Whole blood analyses were performed to determine the leucogram (total leucocytes, neutrophils, lymphocytes, monocytes, eosinophils, basophils) and erythrogram (total erythrocytes, haemoglobin, haematocrit, mean corpuscular volume (MCV), mean corpuscular haemoglobin (MCH), mean corpuscular haemoglobin concentration (MCHC), red cell distribution width (RDW), as well a total platelets and mean platelet volume (MPV).

The serum samples were used to analyse uric acid (UA), gamma-glutamyl transferase (GGT), iron, lactate dehydrogenase, total proteins, alanine aminotransferase (ALT), aspartate aminotransferase (AST), alkaline phosphatase (ALP), glucose, creatinine, urea, magnesium, calcium, phosphorus, cholesterol, and triglycerides.

These samples were transported immediately after collection to the laboratory for further analysis.

### 2.7. Statistical Analysis

Data were analyzed using the statistical package JMP^®^ Pro 17.1.0 by 2023 SAS Institute Inc. © (Cary, NC, USA). The individual piglet was considered the experimental unit for weight determinations and blood parameters (*n* = 48). The pen was considered the experimental unit for feed intake, conversion ratio, and nutrient digestibility (*n* = 24). All parameters were analyzed using analysis of variance (ANOVA), considering treatment as the main source of variation. In addition, a mixed model including pen as a random effect was performed as a sensitivity analysis for body weight and blood parameters, and the conclusions remained unchanged. When significant differences were detected (*p* < 0.05), means were separated using Tukey’s multiple range test at a 5% significance level. Results are expressed as the mean and standard error of the mean (SEM).

## 3. Results

### 3.1. Chemical Composition of BSF Defatted Meal and Diets

The chemical composition of the defatted BSF meal is shown in Table 1. The ingredient showed a high dry matter content (91.3%), with organic matter accounting for 88.3% and ash content of 11.7%. Crude protein concentration reached 45.2%, whereas ether extract was 6.8%, confirming the defatted nature of the insect meal.

Regarding fiber fractions, neutral detergent fiber was not determined; however, acid detergent fiber and acid detergent lignin contents were 13.8% and 7.6%, respectively. These values indicate the presence of structural components typically associated with the insect exoskeleton.

Overall, the chemical profile observed for the defatted BSF meal is consistent with values reported in the literature for defatted black soldier fly larval meals, characterized by high protein content, reduced lipid concentration, and a measurable proportion of structural fiber fractions.

### 3.2. Zootechnical Performances

**Figure 1:**
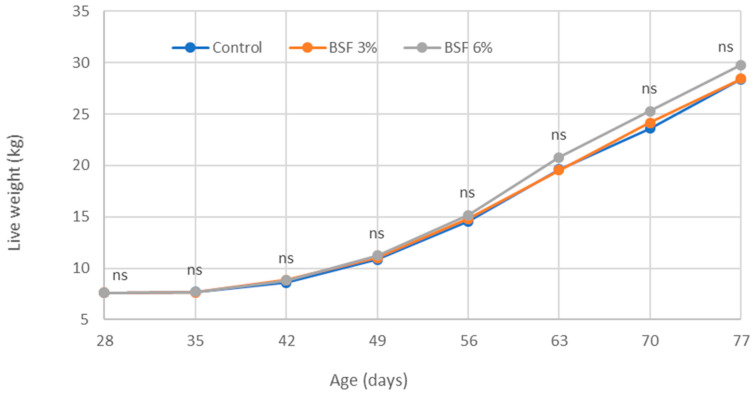
Live weight of animals (kg) during the trial.

Throughout the experimental period, the piglets showed no signs of disease, and mortality was zero in all groups. Figure 1 shows the evolution of the animals’ live weight throughout the experiment. The use of defatted BSF larvae meal had no effect on growth performance (Table 2). There were no significant differences in final body weight, average daily gain, average daily feed intake, or feed conversion ratio between the Control, BSF 3%, and BSF 6% groups (*p* > 0.05), with the exception of feed intake during the 7–28-day period.

The incidence of diarrhea was not affected by dietary treatment during the trial (Table 3).

### 3.3. Digestibility

The inclusion of defatted black soldier fly (BSF) larvae meal in piglet diets influenced nutrient digestibility of dry matter and organic matter (Table 4). Piglets fed the 6% BSF diet showed significantly higher (*p* < 0.05) digestibility of dry matter and organic matter compared with the control and 3% BSF groups. However, no significant differences were observed for crude protein and crude fat digestibility among treatments (*p* > 0.05).

### 3.4. Blood Parameters

The piglets’ haematological and biochemical blood parameters are shown in Table 5. For most parameters, there were no significant differences related to the incorporation of defatted BSF larvae meal in the blood parameters (*p* > 0.05), except for basophils, which increased with the 6% BSF level (*p* < 0.05), with the maximum corresponding to the inclusion of 6% and 3% BSF larvae meal, respectively.

## 4. Discussion

In the present study, the chemical composition of the defatted BSF larval meal showed a high dry matter content and a crude protein concentration of 45.2%, which is within the range commonly reported for black soldier fly larvae meals on a dry matter basis [2,3,18]. These values are consistent with previous reports indicating that BSF larvae typically contain between 40 and 45% crude protein, supporting their suitability as an alternative protein source comparable to conventional ingredients such as soybean meal and fishmeal.

The ether extract content observed in the present study (6.8%) confirms the defatted nature of the insect meal and is lower than values reported for full-fat BSF larvae, which are known to be rich in lipids [2,19]. This reduction is directly associated with the oil extraction process and may provide practical advantages in feed formulation by improving nutrient standardisation and reducing excessive dietary fat inclusion. Moreover, lower lipid levels may help minimise variability associated with fatty acid oxidation and storage stability, which are important considerations for the commercial use of insect-derived ingredients.

The presence of acid detergent fiber and acid detergent lignin in the defatted BSF meal reflects the contribution of structural components associated with the insect exoskeleton, notably chitin, as previously described in the literature [6,22,40,41]. Although neutral detergent fiber was not determined, the observed levels of acid detergent fiber and lignin are consistent with previous studies reporting measurable structural fiber fractions in insect meals. Chitin is generally considered poorly digestible in monogastric animals and may influence nutrient digestibility depending on its concentration and physicochemical properties. However, chitin has also been associated with potential prebiotic and immunomodulatory effects, suggesting that its role in animal nutrition may extend beyond its classification as a structural carbohydrate.

Overall, the chemical profile observed in the present study aligns with previously published data on BSF larval meals, confirming their high protein content, reduced lipid concentration following defatting, and the presence of structural fiber components. These characteristics reinforce the potential of defatted BSF meal as a nutritionally valuable and sustainable protein source for animal feeding systems, as highlighted in earlier studies [15,16,17].

The results suggest that piglets fed diets containing 3% or 6% defatted BSF larvae meal, partially replacing soybean, fishmeal, or both, showed no significant differences in growth performance (ADG, ADFI, FCR, final body weight) compared to the control group, with the exception of feed intake that differed during the 7–28-day period. When soybean was partially replaced by BSF meal, Biasato et al. [33] also reported no significant effects on the zootechnical performance of pigs fed up to 10% defatted BSF meal. Similarly, Driemeyer [41] found that including 3.5% BSF larvae meal in place of soybean did not result in significant differences in feed consumption or weight gain compared to the control group, indicating that BSF larvae meal at this inclusion level supports pig growth similarly to conventional protein sources.

In studies where fishmeal was replaced by BSF larvae meal, ref. [42] reported no impact on average daily feed intake or average daily gain at different replacement levels (0%, 25%, 50%, 75%, and 100%). Sprangher et al. [11] also observed that the use of BSF larvae did not result in significant changes in pigs’ weight gain or feed efficiency. Yu et al. [14] reported a positive effect on daily weight gain and final weight when inclusion was up to 4%, but not at 8%, suggesting that factors such as diet composition, larval stage, and environmental conditions can influence the response.

When both soybean and fishmeal were simultaneously replaced by BSF larvae meal, Phaengphairee et al. [37] observed improvements in average daily gain and feed efficiency, particularly when combined with probiotics, suggesting that additives can optimize nutrient utilisation from this alternative protein source.

No significant differences (*p* > 0.05) were observed among treatments regarding diarrhea scores throughout the experimental period. These results indicate that the inclusion of *Hermetia illucens* meal at 3% and 6% did not negatively affect gastrointestinal health or fecal consistency in piglets. Overall, diarrhea scores remained close to 1 across all treatments, indicating that most feces were classified as normal during the experimental period. This suggests that piglets maintained good gut health regardless of dietary treatment.

Recent studies have highlighted the potential role of insect-derived ingredients in promoting intestinal health in pigs [11]. Insects may represent promising functional feed ingredients due to the immunostimulatory properties of chitin, as well as the antibacterial and antimicrobial activities of antimicrobial peptides and lauric acid, which may collectively contribute to enhanced intestinal stability and reduced incidence of digestive disturbances [6,37]. Therefore, the inclusion of BSF meal may provide functional benefits beyond its nutritional contribution alone.

Regarding blood parameters, the present study showed no significant differences in the erythrogram and leukogram. Although the number of basophils differed significantly among treatments, all values remained within the physiological reference range reported for pigs (0–2%) according to Weiss and Wardrop [43], suggesting the absence of pathological or inflammatory conditions. Basophils are associated with immune and inflammatory responses, and slight variations in these cells may occur as a consequence of dietary changes. In the present study, however, the low basophil counts indicate that the observed differences were not biologically relevant. These findings are consistent with [33], who reported no adverse effects on the haematological parameters of pigs fed up to 10% defatted BSF meal. Similarly, Chia et al. [42] observed no significant differences in red and white blood cell indices between dietary treatments, except for platelets and neutrophils, which showed statistical differences.

Dietary inclusion of defatted BSF larvae meal did not affect blood biochemical parameters such as cholesterol, triglycerides, LDH, and HDL. Yu et al. [14] observed a positive effect on leukocyte count, focusing on lymphocytes. Phaengphairee et al. [37] reported increased immunoglobulin A and glutathione peroxidase activity, indicating positive modulation of the immune system. Driemeyer et al. [41] observed no significant differences in blood and biochemical parameters (albumin, calcium, phosphorus, iron, IgA, IgC, and IgM) when pigs were fed 3.5% whole BSF meal in place of soybean.

In the present study, piglets fed the diet containing 6% defatted BSF larvae meal (BSF 6%) showed significantly higher dry matter (80.3% ^a^) and organic matter digestibility (82.5% ^a^) compared to the control (DM: 78.7% ^b^; OM: 81.3% ^ab^) and BSF 3% (DM: 78.2% ^b^; OM: 81.3% ^b^) treatments (*p* < 0.05), indicating that higher inclusion levels of BSF meal may enhance nutrient utilisation, whereas lower inclusion levels did not produce similar improvements. Although BSF meal contains higher levels of structural components such as chitin and fibre fractions (ADF and ADL), which are generally associated with reduced digestibility, previous studies in piglets have reported that the inclusion of insect meals does not necessarily impair nutrient digestibility and may, in some cases, maintain or improve digestive efficiency. In particular, ref. [11] reported that the inclusion of BSF meal in diets for weaned piglets did not negatively affect nutrient digestibility and supported efficient nutrient utilisation. Similarly, Biasato et al. [33] observed no significant differences in nutrient digestibility parameters, while [35] reported that the inclusion of up to 10% BSF meal in piglet diets had no detrimental effects on digestibility. In contrast, ref. [37] observed improved digestibility following the inclusion of BSF larvae meal, particularly when combined with probiotics, resulting in enhanced nutrient retention, greater digestibility, and reduced inflammatory markers.

These responses may be explained by functional effects of insect-derived ingredients on the gastrointestinal tract. It has been suggested that chitin and chitosan may exert prebiotic-like effects, contributing to modulation of the intestinal microbiota and improvement of gut health, which can enhance nutrient utilisation despite higher fibre content. In addition, recent reviews on insect-based feeds in swine nutrition [42], indicate that such functional changes may offset the potential negative effects of structural carbohydrates on digestibility. Therefore, the improved digestibility observed at 6% inclusion level may reflect a combined effect of diet composition and microbiota-mediated functional responses rather than a simple effect of nutrient composition alone.

In summary, the replacement of soybean, fishmeal, or both by BSF larvae meal was feasible in piglet diets, maintaining growth performance, blood parameters within physiological ranges, and good nutrient digestibility, highlighting the potential of BSF meal as a sustainable alternative protein source with practical applications in swine production.

## 5. Conclusions

In conclusion, the inclusion of defatted *Hermetia illucens* larvae meal at levels up to 6% in diets for weaned piglets did not negatively affect growth performance or health status, indicating its suitability as a partial alternative protein source in swine nutrition.

The blood parameters remained within the normal physiological ranges, confirming the absence of adverse effects on animal health.

Nutrient digestibility results showed improved dry matter and organic matter utilisation at the highest inclusion level, while crude protein and fat digestibility were not affected, suggesting a potential functional effect of insect-based ingredients on nutrient utilisation.

Overall, these findings support the use of defatted BSF meal as a sustainable feed ingredient capable of partially replacing conventional protein sources such as soybean meal and fishmeal without compromising performance. However, further studies are required to evaluate higher inclusion levels, long-term effects, and the underlying mechanisms involved, particularly those related to gut health and microbial modulation.

## Figures and Tables

**Table 1:** Ingredients, chemical composition, minerals and amino acids of BSF feed and defatted meal.

Animals Age (Days)		Phase 1—Pre-Starter (PS)			Phase 2—Starter (S)		
		(35–56)			(57–77)		
	BSF Meal	Control	BSF 3%	BSF 6%	Control	BSF 3%	BSF 6%
Ingredients (g/100 g)							
MIX	-	75.0	75.0	75.0	75.0	74.5	75.5
R mix PS	-	12.6	12.2	12.5	-	-	-
R mix S	-	-	-	-	3.4	3.4	3.3
Soya meal 48	-	3.0	3.0	-	15.2	15.5	11.7
Wheat	-	3.0	3.0	3.0	-	-	-
Fish meal 70	-	2.5	-	-	2.5	-	-
BSF defatted meal	-	-	3.0	6.0	-	3.0	6.0
Lactose	-	2	2	2	-	-	-
Soya oil	-	2.86	2.7	2.3	2.04	1.99	1.85
Salt	-	-	-	-	2.7	1.8	2.4
(g/kg DM)	Chemical composition (determined)						
Dry matter	91.3	88.4	88.5	88.6	88.4	88.1	88
Ash	11.7	5.4	4.5	4.6	5.2	4.8	4.8
Organic matter	88.3	94.6	95.5	95.4	94.8	95.2	95.2
NDF	-	15.9	15.9	15.7	15.5	14.9	16.9
ADF	13.8	4.3	4.7	5.6	4.5	5	5.1
ADL	7.6	0.9	1.6	1.6	1.2	1.1	1.1
Crude protein	45.2	15	15.1	15.4	19.8	20.4	19.4
Crude fat	6.8	5	5.4	5.1	5.7	5	4.4
(g/100 g)	Macrominerals (determined)						
Magnesium	0.3359	0.1479	0.1287	0.1323	0.1483	0.1944	0.1788
Calcium	1.7413	0.8726	0.5831	0.6175	0.5436	0.4631	0.5752
Potassium	3.0655	0.9929	0.9311	0.9374	1.1280	1.4414	1.3509
Sodium	0.3620	0.2455	0.1980	0.1895	0.3515	0.3965	0.3010
(g/100 g)	Microminerals (determined)						
Iron	0.0010	0.0030	0.0055	0.0020	0.0025	0.0020	0.0025
Copper	0.0024	0.0277	0.0262	0.0275	0.0111	0.0145	0.0137
Manganese	0.0034	0.0006	0.0006	0.0005	0.0006	0.0013	0.0011
Zinc	0.0167	0.0181	0.0162	0.0161	0.0147	0.0155	0.0168
(mmol/g of dried weight)	Amino acids essential (estimated)						
Lysine	-	0.0972	0.0972	0.0972	0.0889	0.0889	0.0889
Methionine + Cysteine	-	0.0457	0.0457	0.0457	0.0429	0.0423	0.0418
Threonine	-	0.0789	0.0789	0.0789	0.0756	0.0756	0.0756
Tryptophan	-	0.0147	0.0147	0.0147	0.0216	0.0196	0.0196
(mmol/g of dried weight)	Amino acids essential (determined)						
Histidine	0.1947	0.0515	0.0700	0.1208	0.0650	0.0758	0.0882
Arginine	0.6534	0.3806	0.4352	0.5475	0.3724	0.3986	0.4193
Threonine	0.0801	0.0614	0.0575	0.076	0.0561	0.0537	0.0599
Valine	0.2514	0.0702	0.1107	0.1547	0.0561	0.0576	0.0742
Lysine	0.5473	0.5210	0.5013	0.3560	0.3026	0.3453	0.3169
l-leucine	0.1251	0.0403	0.0486	0.0794	0.0447	0.0443	0.0578
Leucine	0.2302	0.0889	0.0965	0.1660	0.0964	0.0945	0.1172
Phenylalanine	0.0857	0.0332	0.038	0.0551	0.0379	0.036	0.0446
(mmol/g of dried weight)	Amino acids non-essential (determined)						
Aspartic acid + Asparagine	0.3174	0.1125	0.1176	0.2105	0.1127	0.1252	0.147
Serine	0.2838	0.0464	0.0795	0.1179	0.08	0.0706	0.0986
Glutamic acid + Glutamine	0.2838	0.1483	0.1575	0.2252	0.1761	0.1698	0.193
Glycine	0.1118	0.0057	0.0255	0.0657	0.0194	0.0292	0.0478
Alanine	0.2484	0.0896	0.0871	0.1754	0.0812	0.0694	0.1145
Proline	0.18	0.0706	0.0708	0.1419	0.0833	0.0741	0.0999

MIX (g/100 g)—wheat (37.0) + extruded mixture (wheat, corn, rice, flax seeds, barley) (26.6) + barley (20.0) + corn (16.0) + toxin binder (0.3) + antioxidant (0.01). R mix PS—Premix + flax seeds + cookies meal + calcium monophosphate + fibers mixture + lysine + valine + threonine + methionine + tryptophan + toxin binder. R mix S—Premix + calcium monophosphate + calcium carbonate + fibers mixture + lysine + threonine + methionine + tryptophan + toxin binder.

**Table 2:** Performances of piglets fed defatted BSF meal.

Parameter	Diets			SEM	*p*-Value
	Control	BSF 3%	BSF 6%		
Live weight (g)					
Day 0	7620.0	7628.0	7615.0	169.3	0.169
Day 7	7687.5	7640.6	7715.6	207.1	0.967
Day 28	14,584.4	14,812.5	15,193.8	399.4	0.556
Day 49	28,375.0	28,425.0	29,793.8	663.9	0.241
Average weight gain (g/d)					
7–28 days	328.4	341.5	356.1	13.63	0.374
28–49 days	658.4	657.1	697.6	20.10	0.289
7–49 days	493.4	499.3	526.9	11.47	0.113
Average daily feed intake (g/d)					
7–28 days	488.1 ^b^	536.2 ^a^	504.2 ^ab^	10.53	0.013
28–49 days	1097.1	1100.6	1168.4	24.60	0.093
7–49 days	792.6	818.4	836.3	14.70	0.132
Feed conversion ratio					
7–28 days	1.51	1.60	1.42	0.07	0.231
28–49 days	1.67	1.68	1.68	0.04	0.966
7–49 days	1.61	1.64	1.59	0.02	0.329

Control—control diet; BSF 3%—3% defatted BSF larval meal; BSF 6%—6% defatted BSF larval meal; SEM: standard error of the mean Values are presented as mean. Significant differences were considered at *p* < 0.05. Different superscript letters within a row indicate significant differences among treatments (Tukey’s test, *p* < 0.05).

**Table 3:** Average values of diarrhea scoring.

Treatment	Week 1	Week 2	Week 3	Week 4	Week 5	Week 6	Total Period
Control	1.10	1.09	1.53	1.22	1.26	1.13	1.22
BSF 3%	1.29	1.10	1.45	1.23	1.49	1.28	1.31
BSF 6%	1.13	1.06	1.39	1.34	1.20	1.04	1.19
*p* value	0.144	0.773	0.784	0.469	0.282	0.168	0.476
St Error	0.056	0.038	0.142	0.079	0.134	0.088	0.068

Diarrhea scoring: 1 = Normal, 2 = Pasty, 3 = Semi-liquid, 4 = Liquid.

**Table 4:** Effects of defatted black soldier fly (BSF) larvae meal on the apparent total tract digestibility.

Parameters	Experimental Treatments			Std Error	*p*-Value
	Control	BSF 3%	BSF 6%		
Dry Matter (DM)	78.7 ^b^	78.2 ^b^	80.3 ^a^	0.350	0.0030
Organic Matter (OM)	81.3 ^ab^	81.3 ^b^	82.5 ^a^	0.308	0.0058
Crude Protein (CP)	68.1	69.6	71.9	0.979	0.0952
Crude Fat (CF)	69.1	72.8	76.2	2.891	0.5591

Control—control diet; BSF 3%—3% defatted BSF larval meal; BSF 6%—6% defatted BSF larval meal; Values are presented as means. Significant differences were considered at *p* < 0.05. Different superscript letters within a row indicate significant differences among treatments (Tukey’s test, *p* < 0.05).

**Table 5:** Effects of including defatted BSF larvae meal on the haemogram and blood biochemistry of piglets in the rearing phase.

Parameters		Experimental Treatments			Std Error	*p*-Value
		Control	BSF 3%	BSF 6%		
Leucogram	Total leucocytes (×10^3^/uL)	20.90	19.80	21.10	1.2123	0.6841
	Neutrophils (%)	30.90	30.20	30.30	1.8213	0.9248
	Lymphocytes (%)	63.30	64.20	63.20	1.8213	0.9248
	Monocytes (%)	4.70	4.60	5.30	0.3139	0.3060
	Eosinophils (%)	0.70	0.70	0.60	0.0865	0.4815
	Basophils (%)	0.40 ^ab^	0.30 ^b^	0.60 ^a^	0.0841	0.0211
Erythogram	Total erythrocytes (×10^6^/uL)	6.30	6.20	6.30	0.1336	0.9879
	Haemoglobin (g/dL)	10.80	10.80	10.70	0.2402	0.9290
	Haematocrit (%)	35.30	35.20	34.70	0.8473	0.8642
	MCV (fL)	56.30	56.50	55.50	0.5932	0.4198
	MCH (pg)	17.30	17.40	17.10	0.1621	0.6382
	MCHC (g/dL)	30.70	30.80	30.90	0.2013	0.7802
	RDW (%)	23.00	23.00	22.10	0.4488	0.2574
	Total platelets (×10^3^/uL)	314.00	259.30	257.50	42.4235	0.5680
Biochemistry	GGT (U/L)	43.10	43.30	50.70	3.8440	0.2890
	Iron (ug/dL)	160.90	196.90	157.90	14.6800	0.1230
	LDH (U/L)	1031.00	979.00	1117.00	145.7000	0.7970
	TPOT (g/dL)	5.00	5.00	5.20	0.0660	0.2800
	ALT	41.70	42.70	46.80	2.8190	0.4030
	AST (U/L)	121.50	80.30	122.40	32.2300	0.5770
	ALP (mg/dL)	277.10	294.30	277.90	12.5500	0.5550
	Glucose (mg/dL)	115.70	117.90	113.70	3.0820	0.6230
	Creatinine (mg/dL)	0.70	0.60	0.60	0.0480	0.4016
	Urea (mg/dL)	10.40	7.90	9.50	0.1710	0.9120
	Magnesium (mg/dL)	2.90	3.00	3.00	0.0960	0.6510
	Calcium (mg/dL)	13.00	12.30	13.00	0.4300	0.4090
	Phosphorus (mg/dL)	12.70	13.20	12.9	0.4820	0.7034
	Cholesterol (mg/dL)	87.50	90.00	96.10	3.7040	0.2520
	Triglycerides (mg/dL)	43.70	51.40	56.10	5.2640	0.2550

Control—control diet; BSF 3%—3% defatted BSF larval meal; BSF 6%—6% defatted BSF larval meal; MCV—Mean Corpuscular Volume; MCH—Mean Corpuscular Haemoglobin; MCHC—Mean Corpuscular Haemoglobin Concentration; RDW—Red Cell Distribution Width; GGT—Gamma-Glutamyl Transferase; LDH—Lactate Dehydrogenase; TPOT—Total Protein; Alanine Aminotransferase; AST—Aspartate Aminotransferase; ALP—Alkaline Phosphatase. Values are presented as mean. Significant differences were considered at *p* < 0.05. Different superscript letters within a row indicate significant differences among treatments (Tukey’s test, *p* < 0.05).

## Data Availability

The data that support the findings of this study are available from the corresponding author, M.P., upon reasonable request.
